# N6-Methyladenosine Methylation Analysis of Long Noncoding RNAs and mRNAs in IPEC-J2 Cells Treated With *Clostridium perfringens* beta2 Toxin

**DOI:** 10.3389/fimmu.2021.769204

**Published:** 2021-11-22

**Authors:** Jiaojiao Yang, Qiaoli Yang, Juanli Zhang, Xiaoli Gao, Ruirui Luo, Kaihui Xie, Wei Wang, Jie Li, Xiaoyu Huang, Zunqiang Yan, Pengfei Wang, Shuangbao Gun

**Affiliations:** ^1^ College of Animal Science and Technology, Gansu Agricultural University, Lanzhou, China; ^2^ College of Animal Science and Technology, Northwest A&F University, Xian, China; ^3^ Gansu Research Center for Swine Production Engineering and Technology, Lanzhou, China

**Keywords:** M6A methylation, MeRIP-seq, RNA-seq, IPEC-J_2_, CPB_2_

## Abstract

**Background:**

The n6-methyladenosine (m6A) modification is present widely in mRNAs and long non-coding RNAs (lncRNAs), and is related to the occurrence and development of certain diseases. However, the role of m6A methylation in *Clostridium perfringens type C* infectious diarrhea remains unclear.

**Methods:**

Here, we treated intestinal porcine jejunum epithelial cells (IPEC-J2 cells) with *Clostridium perfringens* beta2 (CPB2) toxin to construct an *in vitro* model of *Clostridium perfringens type C (C. perfringens type C)* infectious diarrhea, and then used methylated RNA immunoprecipitation sequencing (MeRIP-seq) and RNA sequencing (RNA-seq) to identify the methylation profiles of mRNAs and lncRNAs in IPEC-J2 cells.

**Results:**

We identified 6,413 peaks, representing 5,825 m6A-modified mRNAs and 433 modified lncRNAs, of which 4,356 m6A modified mRNAs and 221 m6A modified lncRNAs were significantly differential expressed between the control group and CPB2 group. The motif GGACU was enriched significantly in both the control group and the CPB2 group. Gene ontology (GO) and Kyoto Encyclopedia of Genes and Genomes (KEGG) annotation analysis showed that the differentially methylated modified mRNAs were mainly enriched in Hippo signaling pathway and Wnt signaling pathway. In addition, the target genes of the differentially m6A modified lncRNAs were related to defense response to virus and immune response. For example, *ENSSSCG00000042575*, *ENSSSCG00000048701* and *ENSSSCG00000048785* might regulate the defense response to virus, immune and inflammatory response to resist the harmful effects of viruses on cells.

**Conclusion:**

In summary, this study established the m6A transcription profile of mRNAs and lncRNAs in IPEC-J2 cells treated by CPB2 toxin. Further analysis showed that m6A-modified RNAs were related to defense against viruses and immune response after CPB2 toxin treatment of the cells. Threem6A-modified lncRNAs, *ENSSSCG00000042575*, *ENSSSCG00000048785* and *ENSSSCG00000048701*, were most likely to play a key role in CPB2 toxin-treated IPEC-J2 cells. The results provide a theoretical basis for further research on the role of m6A modification in piglet diarrhea.

## Introduction

Bacterial diarrhea in piglets, which leads to a decline in piglet survival and seriously affects pig husbandry, is an urgent problem in pig production. *Clostridium perfringens type C* (*C. perfringens type C*) is one of the main pathogenic bacteria causing diarrhea in piglets (1). *C. perfringens type C* attaches to the top of the villi of intestinal epithelial cells and multiplies in the chorionic basement membrane to produce *C. perfringens* beta2 (CPB2) toxin. CPB2 toxin acts directly on the intestine or is absorbed through the mucosa into the blood circulation, damaging intestinal epithelial cells and triggering an intestinal inflammatory response (2–4). Currently, the prevention and treatment of this diarrhea disease relies mainly on vaccines and antibiotics. Although these measures have reduced and controlled the occurrence of diarrhea to some extent, long-term use of antibiotics has led to increased bacterial resistance and resulted in food safety and human health concerns. In addition, mutation of pathogenic bacteria is a continuous challenge for the research and development of vaccines, making the prevention and treatment of piglet diarrhea more difficult. Therefore, identifying molecular markers related to piglet diarrhea resistance and determining its mechanism of action in the resistance to pathogen infection have become important methods to solve this problem.

RNA methylation modification is one of the important areas of epigenetic research, and n6–methyladenosine (m6A) is a common type of modification in mammalian cells (5). M6A is present widely in eukaryotic mRNAs, long non-coding RNAs (lncRNAs), and circular RNAs (circRNAs) (6). The m6A modification regulates protein expression by influencing the processes of mRNA shearing, transport, translation, and degradation, and participates in the process of resisting bacterial and viral infections, inflammatory bowel disease, and immune responses (7–12). With the emergence of methylated RNA immunoprecipitation sequencing (MeRIP-seq) technology, research into m6A methylation modification has entered a stage of rapid development, which has greatly promoted the identification of RNA m6A modifications in different species (13).

LncRNAs are non-coding RNAs with a length of more than 200 nt, which have crucial roles in the occurrence and development of diarrhea caused by pathogen infection in the host. In the process of piglet diarrhea caused by Porcine epidemic diarrhea virus (PEDV) infection, *lncRNA-9606* plays a regulatory role by activating the T cell receptor signaling pathway of the ileal immune system and promoting the production of IgA (14). *LncRNA-EPS* affects the innate immune response by regulating the expression of immune response genes in mouse macrophages (15). Previous studies have shown that *lnc_033304* and *lnc_001066* might be involved in the defense of piglets against *C. perfringens type C* infection by regulating the key genes of the nuclear factor kappa B (NF-κB), mitogen-activated protein kinase (MAPK), and Toll-like receptor signaling pathways (16). However, whether m6A methylation–regulated lncRNAs are involved in the process of piglet resistance to diarrhea infection, and the molecular mechanism of their functions, are currently unclear. Therefore, it is necessary to study the function and mechanism of m6A-modified lncRNAs in the process of piglet diarrhea.

In the present study, a cell model of *C. perfringens type C* infection was constructed, and MeRIP-seq was performed on these cells. We obtained the transcription profiles of m6A-modified mRNAs and lncRNAs in intestinal porcine jejunum epithelial cells (IPEC-J2) treated with CPB2 toxin. The general characteristics, topological patterns, and participation in biological pathways of m6A methylation were compared, thereby providing an improved understanding of the roles of the m6a modification in piglet diarrhea. The results of the present study expand our understanding of the molecular mechanisms underlying the m6A modification in piglet diarrhea.

## Materials and Methods

### CPB2 Toxin Protein Preparation

We used the method described by Gao and Luo (17, 18) to extract and purify the CPB2 toxin protein. Then, the endotoxin was removed using Endotoxin Erasol (Genscript, Nanjing, China). Finally, the integrity of the CPB2 protein was assessed using 12% SDS–PAGE.

### Cell Culture and Treatment

IPEC-J2 cells were purchased from the BeNa Culture Collection (BNCC, Beijing, China) and cultured in Dulbecco’s modified Eagle’s medium (DMEM, Grand island, NY, USA)/F12 medium (Gibco), to which 10% FBS (fetal bovine serum; GeminiBio, Sacramento, CA, USA) and antibiotics (penicillin and streptomycin) were added; the cells were maintained at 37°C in a 5% CO_2_ atmosphere. IPEC-J2 cells were stimulated with CPB2 (20 μg/mL) for 24 h, which was defined as the CPB2 group, and unstimulated IPEC-J2 cells were defined as the control group.

### Total m6A Measurement

Total RNA was extracted using the TRIzol™ reagent (Invitrogen, Carlsbad, CA, USA). An EpiQuik m6A RNA methylation quantification kit (colorimetric method) (AmyJet Scientific, Wuhan, China) was then used to determine the total m6A content.

### MeRIP-Seq and RNA-Seq

Total RNA from IPEC-J2 cells was isolated using the TRIzol™ reagent. The RNA concentration and integrity of IPEC-J2 cells were estimated using a NanoDrop ND-8000 spectrophotometer (NanoDrop, Wilmington, DE, USA) and an Agilent 2100 Bioanalyzer (Agilent Technologies, Santa Clara, CA, USA) to select high-quality RNA samples [RNA integrity number (RIN) > 7). A Ribo-Zero rRNA Removal Kit (Illumina, San Diego, CA, USA] was used to remove ribosomal RNA from the total RNA exceeding 25 μg. Then, these RNAs were fragmented into small fragments using a Magnesium RNA Fragmentation Module (NEB, Ipswich, MA, USA; cat no. e6150). Half of the approximately 100 nt fragmented RNAs were used for immunoprecipitation (IP), and the other half was used as the IP control (Input). Then, the cleaved RNA fragments were incubated with an m6A-specific antibody (No. 202003, Synaptic Systems, Göttingen, Germany) to obtain IP RNA, which was then reverse transcribed to generate cDNA, end repaired, added with an A tail and connectors, purified, and PCR enriched, all performed according to the manufacturer’s instructions. MeRIP-Seq and RNA-seq libraries (IP and Input) were then constructed, and 2 × 150 bp paired-end sequencing (PE150) was performed on an Illumina Novaseq™ 6000 instrument by LC-Bio Technology CO., Ltd. (Hangzhou, China).

### Bioinformatic Analysis Process

Fstp software was used to perform quality control on the original data from the IP and Input samples, including removing connectors, repeated sequences, and low-quality sequences (quality scores < Q20) to obtain clean data (19). The clean reads were mapped to the pig reference genome sequence (*Sus scrofa* 11.1 v101) using HISAT2 (20). The R package exomePeak was used to perform peak calling analysis and genetic difference peak analysis on the IP and Input samples, and then we used ChIPseeker to annotate the peaks (21). Finally, MEME2, HOMER, and ChIPseeker software were used for motif analysis, positioning, and annotation (22–24). StringTie software was used for gene assembly and quantification *via* FPKM (fragments per kilobase of exon model per million mapped reads) (25).

Transcriptomes from the samples were merged to reconstruct a comprehensive transcriptome using gffcompare (26). First, we removed the known mRNAs and transcripts smaller than 200 bp, and then used CPC (27) and CNCI (28) to predict the coding potential of the remaining transcripts, filtering out the transcripts with CPC scores < 1 and CNCI scores < 0 to obtain lncRNAs. Significantly differentially expressed mRNAs and lncRNAs were selected using |log2 (fold change)| ≥ 1.0 and *P*-value < 0.05 in the R package edgeR (29).

*Cis* and *trans* methods were used to analyze and predict the target genes of the lncRNAs. The cis-regulated target genes were derived from differential expressed mRNAs in the range of 100 kb upstream and downstream of the lncRNA (30). The trans-regulatory target genes were identified using RIsearch (36.3) software (31). The free energy of forming a secondary structure between an lncRNA and an mRNA sequence should be lower than the threshold value of -11, and they should also show expression correlation (the absolute value of Pearson correlation coefficient should be greater than 0.95). Finally, Gene Ontology (GO) (http://www.geneontology.org/) and Kyoto Encyclopedia of Genes and Genomes (KEGG) (http://www.kegg.jp/) enrichment analysis were used to annotate the functions of the lncRNA target genes (32, 33).

### MeRIP-qPCR

M6A-modified RNAs from the IP sample and RNAs from the Input sample were reverse–transcribed into cDNA using a reverse transcriptase kit (TransGen Biotech, Beijing, China). Quantitative real-time polymerase chain reaction (qPCR) was performed using the cDNA as the template on a LightCycler 480 instrument (Roche Applied Science, Mannheim, Germany) with the SYBR Green Realtime PCR Master Mix (Accurate Biotechnology, Hunan, China). Glyceraldehyde-3-phosphate dehydrogenase (*GAPDH)* was used as an internal control. The primer information is presented in **Supplementary Table 1**. The relative expression levels were calculated using the 2^−ΔΔCt^ method (34).

### Statistical Analysis

SPSS v.20 software (IBM Corp, Armonk, NY, USA) and GraphPad Prism 6.0 software (GraphPad Inc., La Jolla, CA, USA) were used for all statistical analyses. Data are displayed as the mean ± SD. The analysis was performed using Student’s t test (two-tailed). *P* < 0.05 (*) and *P* < 0.01 (**) were considered statistically significant.

## Results

### The Total m6A Content in IPEC-J2 Cells

**Figure 1:**
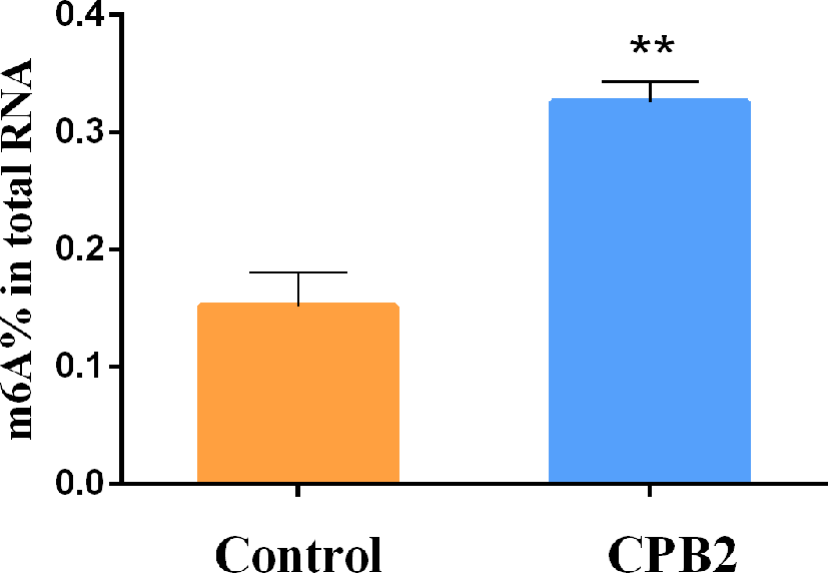
The total m6A content in the CPB2-treated IPEC-J2 cells ** means P < 0.01.

To explore whether CPB2 toxin-treated cells were related to m6A methylation, the overall level of m6A methylation was determined. These results showed that the overall level of m6A methylation increased significantly after CPB2 toxin treatment (**Figure 1**). Therefore, we speculated that the m6A modification was involved in the physiological changes of IPEC-J2 cells after CPB2 treatment.

### Characteristics of the Sequencing Data

Transcriptome sequencing constructed 12 libraries from six samples, with more than 82,300,026 clean reads per sample in MeRIP-Seq data. The ratio of Q30 (sequencing error rate less than 0.001) for all samples ranged from 90.59 to 91.17%. The average GC content of the twelve libraries was 54.41%. (**Supplementary Table 2**). About 80.00% of the clean reads could be aligned to the pig reference genome sequence (Sus scrofa 11.1), with above 60.00% of the reads being uniquely aligned (**Supplementary Table 3**). According to the regional information of the pig reference genome, clean reads that can be matched to the reference genome were defined as exons, introns and intergenic sequences. In the control group, the average proportions of exons, introns, and intergenic sequences of IPEC-J2 cell IP samples were 75.06%, 21.39%, and 3.55%, and were 69.77%, 27.84%, and 2.39%, respectively, in the input samples. In addition, after CPB2 toxin treatment, the average proportions of exon, intron, and intergenic sequences in the IP samples from IPEC-J2 cells were 73.20%, 23.34%, and 3.46%, respectively, and were 64.62%, 32.74%, and 2.64%, respectively, in the Input samples (**Supplementary Table 4**).

### General Features of IPEC-J2 Cell m6A Methylation

**Figure 2:**
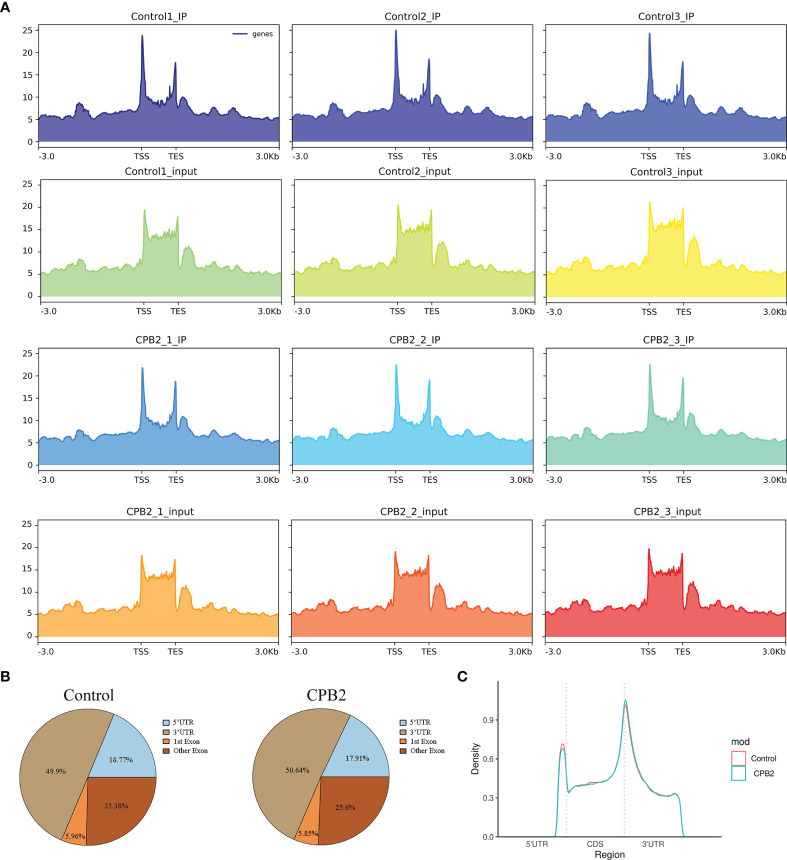
General features of IPEC-J2 cells m6A methylation. **(A)** The enrichment of peaks near the gene transcription start site. **(B)** The position distribution of m6A peaks on gene functional elements. **(C)** The m6A peak density along a metagene.

The peak-calling software, R package exomePeak, was used to perform peak scanning of the entire gene range. The peak identification data came from MeRIP-seq sequencing data. Regions with a P value < 0.05 were considered as peaks. All samples were combined to show the enrichment of peaks distribution near the gene transcription start site (TSS) (**Figure 2A**). A total of 6,413 peaks were simultaneously identified in the two groups. Among them, there were 1,752 significantly different peaks, identified using screening conditions of |log2 (fold change) | ≥ 1, and P < 0.05. Compared with the control group, 521 peaks were upregulated in the CPB2 group involved 78 differentially expressed genes, and 1,231 peaks were downregulated in the CPB2 group involved 143 differentially expressed genes (**Supplementary Table 5**). The m6A modification can affect mRNA transcription, splicing, localization, translation, stability, and post-transcriptional regulation at the RNA level. To further understand the preferential position of m6A on the transcripts, we divided the transcripts into four areas: 5’ untranslated region (5’ UTR), 1st exon, other exon, and 3′ UTR (**Figure 2B**). We found that the differential m6A peaks were mainly enriched in the 3′ UTRs. At the same time, the peak density further confirmed that m6A was enriched around the 3’ UTR (**Figure 2C**). The top 20 significantly different m6A modified peaks are shown in **Table 1**.

**Table 1:** The top 20 significantly differentially expressed m6A peaks.

Gene Name	log2_fold change	Regulation	Chromosome	Peak region	Peak star	Peak end	P-value
*ENSSSCG00000031620*	6	Hyper-methylation	chr12	3’ UTR	41052313	41075862	0.00
*FZD9*	5.68	Hyper-methylation	chr3	Exon	10754783	10757147	0.00
*ENSSSCG00000033352*	5.17	Hyper-methylation	chr9	Exon	121380415	121461054	0.02
*ENSSSCG00000033909*	5.11	Hyper-methylation	chr18	Exon	6372382	6386982	0.00
*ENSSSCG00000034356*	4.89	Hyper-methylation	chr6	Exon	10404926	10407641	0.00
*ENSSSCG00000042192*	4.18	Hyper-methylation	chr14	5’ UTR	48609742	48611583	0.00
*TNFAIP8L2*	4.13	Hyper-methylation	chr4	3’ UTR	98091704	98096514	0.01
*ENSSSCG00000013607*	4.04	Hyper-methylation	chr2	Exon	70621910	70623031	0.03
*D2HGDH*	4.04	Hyper-methylation	chr15	Exon	140282133	140322624	0.00
*UNC93B1*	3.91	Hyper-methylation	chr2	3’ UTR	4855425	4909856	0.03
*ENSSSCG00000034853*	-10.20	Hypo-methylation	Y	3’ UTR	25246203	25253238	0.00
*UGGT2*	-4.85	Hypo-methylation	chr11	5’ UTR	65307150	65476547	0.00
*ENSSSCG00000042133*	-4.64	Hypo-methylation	chr12	Exon	3974787	3981980	0.00
*MYO9A*	-4.46	Hypo-methylation	chr1	3’ UTR	169373888	169596636	0.05
*IQGAP2*	-4.46	Hypo-methylation	chr2	Exon	85294434	85636317	0.02
*TASP1*	-4.13	Hypo-methylation	chr17	Exon	21964431	22357413	0.01
*ENSSSCG00000041170*	-4.01	Hypo-methylation	chr6	Exon	169215702	169221363	0.01
*SV2A*	-3.94	Hypo-methylation	chr4	5’ UTR	99138342	99153149	0.00
*ENSSSCG00000042741*	-3.86	Hypo-methylation	chr4	Exon	122621045	122757332	0.01
*ENSSSCG00000042951*	-3.85	Hypo-methylation	chr11	Exon	6943191	6949778	0.00

### Motif Analysis

**Figure 3:**
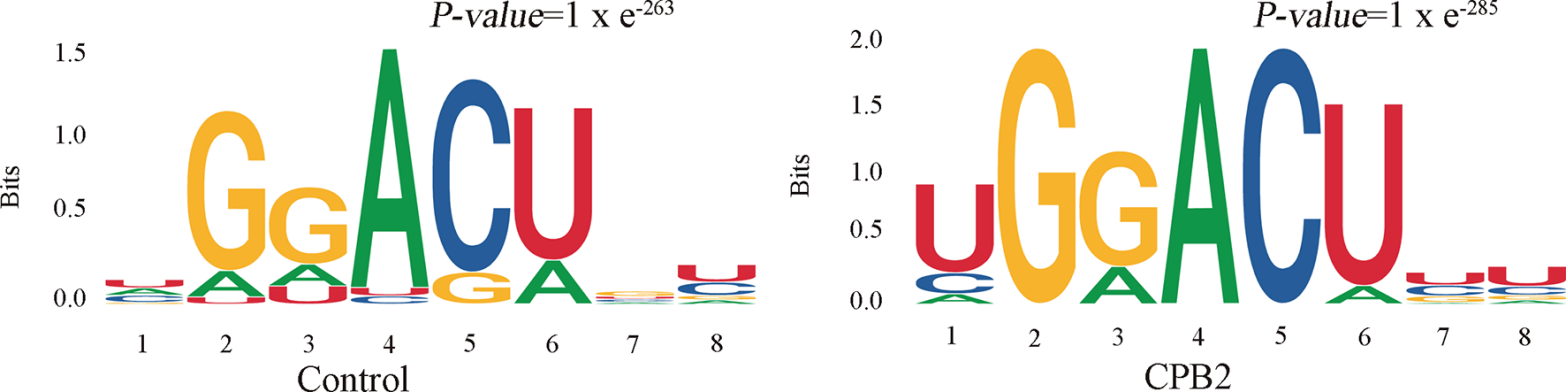
The RRACH conserved sequence motif for m6A peak regions.

Motifs are nucleic acid sequence patterns with biological significance, and RNA methylation–related enzymes can recognize and bind these motifs to regulate gene expression. To determine whether the identified m6A peaks contained the RRACH (R = A or G, H = A, C or U) conserved sequence motif, HOMER (motif analysis software) was used to find credible motifs in the peak areas. These results showed that both the control group and the CPB2 group contained the motif GGACU (**Figure 3**).

### Differential Expression of mRNAs and lncRNAs

**Figure 4:**
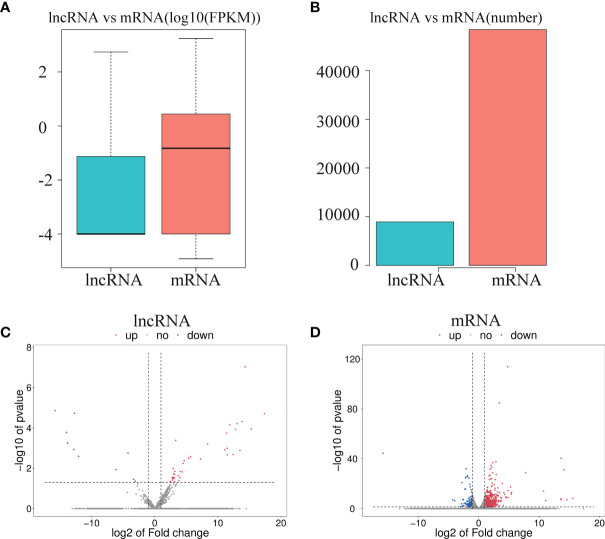
The differential expression of lncRNAs and mRNAs. **(A)** The significantly differentially expressed lncRNAs and mRNAs between the CPB2 group and the control group (using the log10 FPKM method) box plots. **(B)** The number of significantly differentially expressed lncRNAs and mRNAs. **(C)** The differentially expressed lncRNAs in the control group and CPB2 group. **(D)** The differentially expression of mRNAs in the control group and the CPB2 group. Blue indicates a significant increase, and red indicates a significant decrease.

To investigate the effect of CPB2 toxin treatment on IPEC-J2 cells, we analyzed the levels of differentially expressed mRNAs and lncRNAs in the cells between the control group and CPB2 group using the edge R software. The expression level and quantity of mRNAs and lncRNAs are shown in **Figures 4A, B**. The volcano map shows the differentially expressed lncRNAs and mRNAs (**Figures 4C, D**). There were 760 mRNAs and 49 lncRNAs showing significant differential expression between the control group and the CPB2 group. Compared with the control group, 39 lncRNAs and 596 mRNAs were significantly upregulated in the CPB2 group, while 10 lncRNAs and 164 mRNAs were significantly downregulated in the CPB2 group.

### Functional Enrichment Analysis of Differentially Methylated mRNAs

**Figure 5:**
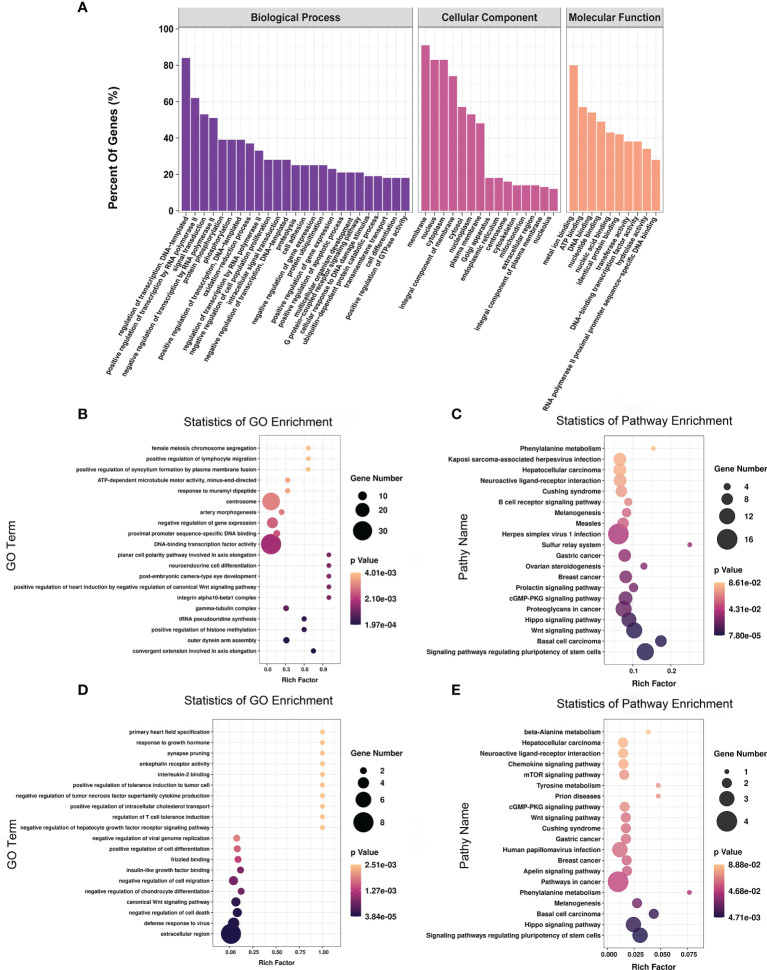
The distribution and functional analysis for differential m6A-modified mRNAs. GO enrichment **(A, B)** and KEGG signaling pathway analysis **(C)** of differential m6A-modified mRNAs. GO enrichment **(D)** and KEGG signaling pathway analysis **(E)** of mRNAs with simultaneous differences in m6A and transcription levels.

To explore the function of m6A-modified mRNAs, GO and KEGG pathway analyses were performed for the 4,356 significantly differentially expressed m6A-modified mRNAs. The screening threshold was |(log2 (fold change)| ≥ 1, and *P* < 0.05. These mRNAs were enriched for 2,801 GO terms and 244 KEGG pathways (**Supplementary Table 6**). The significantly enriched GO terms included defense response to positive regulation of histone methylation and proximal promoter sequence-specific DNA binding (**Figures 5A, B**). KEGG Pathway analysis recognized that the differentially m6A-modified mRNAs were significantly associated with Herpes simplex virus 1 infection, Regulating pluripotency of stem cells, the Wnt signaling pathway, and the Hippo signaling pathway (**Figure 5C**).

To further investigate the effect of m6A-modified mRNAs on CPB2-induced IPEC-J2 cells, we conducted a combined analysis of MeRIP-seq and RNA-seq data. We identified 36 mRNAs with significant differences in their methylation level and transcription level (**Supplementary Table 7**). The screening criteria were |(log2 (fold change)| ≥ 1, and P < 0.05. GO enrichment analysis of these 36 genes found that extracellular region, defense response to virus, and negative regulation of cell death were all significantly enriched terms (**Figure 5D**). In the KEGG analysis, signaling pathways regulating the pluripotency of stem cells, the Hippo signaling pathway, and pathways in cancer were the most significant enriched pathways (**Figure 5E**).

### Functional Enrichment Analysis of Differentially Methylated lncRNAs

**Figure 6:**
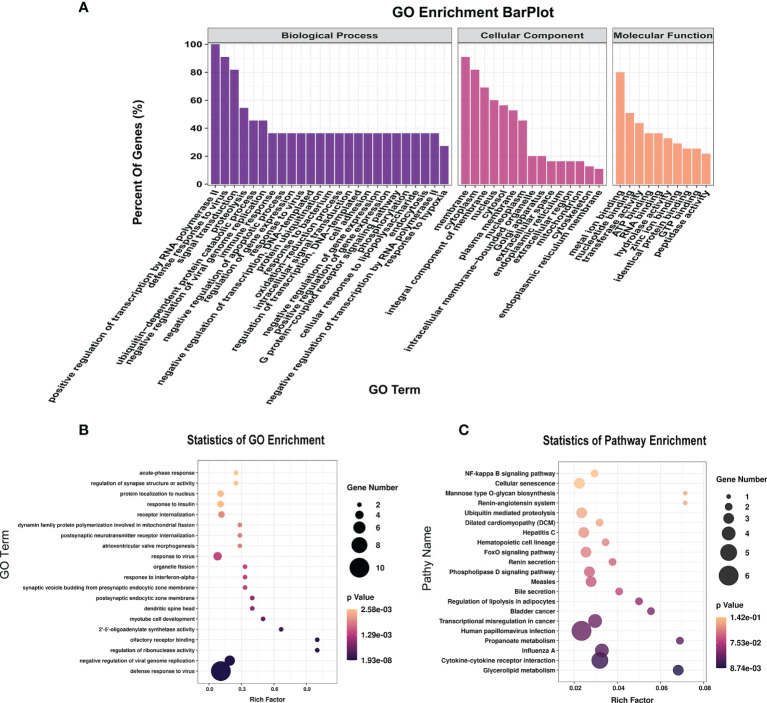
The distribution and functional analysis for differential m6A-modified lncRNAs. GO enrichment **(A, B)** and KEGG signaling pathway analysis **(C)** of the target genes of differentially m6A modified lncRNAs.

To further explore the functions of the 153 significantly differentially expressed m6A-modified lncRNAs, we used the screening conditions |(log2 (fold change)| ≥ 0.585, and *P* < 0.05 to predict the cis–regulated and trans-regulated target genes of these lncRNAs. The results showed that a total of 140 target genes could be predicted (**Supplementary Table 8**), and then GO and KEGG enrichment analyses were used to analyze the target genes. These target genes were enriched for 1,148 GO terms and 166 KEGG pathways. The significantly enriched GO terms included defense response to virus, negative regulation of viral genome replication, response to virus, response to interferon-alpha, and regulation of ribonuclease activity (**Figures 6A, B**). KEGG Pathway analysis showed that the target genes of the differentially expressed m6A-modified lncRNAs were significantly associated with Human papillomavirus infection, Influenza A, Hepatitis C, and the NF-kappa B signaling pathway (**Figure 6C**).

**Figure 7:**
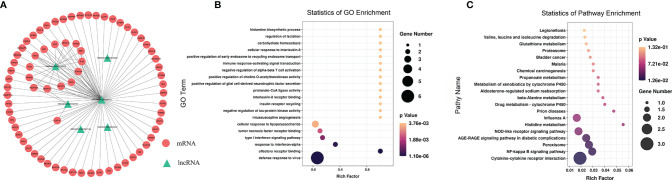
Combined analysis of lncRNAs in the RNA-seq and MeRIP-seq data. The network between differentially expressed m6A-modified lncRNAs and differentially expressed mRNAs **(A)**. The red circles represent significantly differentially expressed mRNAs, and the green triangles represent significantly differentially expressed m6A-modified lncRNAs. GO enrichment **(B)** and KEGG signaling pathway analysis **(C)** of differentially expressed target genes of differentially m6A-modified lncRNAs.

To study the effect of m6A-modified lncRNAs on CPB2-induced IPEC-J2 cells, we performed correlation analysis of the MeRIP-seq and RNA-seq data, and screened out six lncRNAs with the most significant differences in m6A modification level and transcription level (**Supplementary Table 9**). The screening threshold was |(log2 (fold change)| ≥ 0.585, and P < 0.05. Then these six lncRNAs and their corresponding target mRNAs were constructed into an lncRNA-mRNA network (**Figure 7A**). GO enrichment analysis of the target genes of these six lncRNAs found that defense response to virus was the most significant enrichment item, and immune response-activating signal transduction and Interleukin-8 receptor binding were significantly enriched terms (**Figure 7B**). In the KEGG analysis, cytokine-cytokine receptor interaction and NF-kappa B signaling pathway were the most significantly enriched pathways (**Figure 7C**). We found that there was a common differential expression target gene, *CXCL8* (encoding C-X-C motif chemokine ligand 8), in these two pathways (**Supplementary Table 10**).

### Validation of Differentially Expressed lncRNAs and m6A-Modified lncRNAs

**Figure 8:**
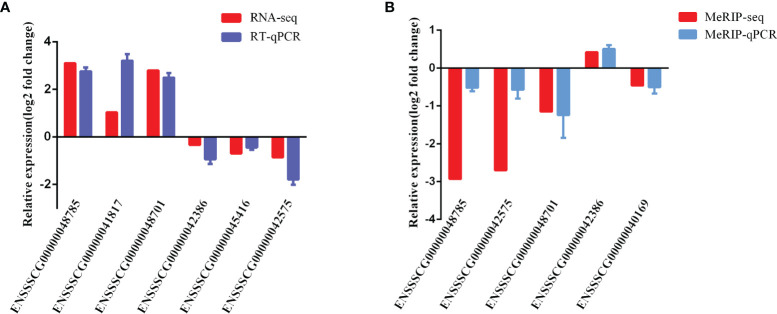
Validation of MeRIP-seq and RNA-seq by qRT-PCR and MeRIP-qPCR. **(A)** RT-qPCR validation of six lncRNAs. **(B)** MeRIP-qPCR validation of five m6A-modified lncRNAs.

To validate the reliability of our RNA-seq and MeRIP-seq results, six differentially expressed lncRNAs and five differentially expressed m6A-modified lncRNAs were selected randomly and subjected to qRT-PCR and MeRIP-qPCR analysis. In the qRT-PCR results, the expression levels of *ENSSSCG00000042386*, *ENSSSCG00000045416*, and *ENSSSCG00000042575* were lower in the CPB2 group than in the control cells, while the expression levels of *ENSSSCG00000048785*, *ENSSSCG00000041817*, and *ENSSSCG00000048701* were higher in the CPB2 group cells than in control cells (**Figure 8A**). In the MeRIP-qPCR results, the expression m6A level of m6A-modified *ENSSSCG00000042386* was increased after CPB2 treated cells, while the expression of m6A-modified *ENSSSCG00000048785*, *ENSSSCG00000042575*, *ENSSSCG00000048701*, and *ENSSSCG00000040169* were reduced after CPB2 treated cells (**Figure 8B**). These results indicated that the regulatory direction and expression pattern of these lncRNAs, as verified by RT-qPCR and MeRIP–qPCR, were consistent with the sequencing results, thus confirming the reliability and reproducibility of RNA-seq and MeRIP-seq methods.

## Discussion

*C. perfringens type C* is a common pathogen of newborn livestock. Animals infected with this pathogen might suffer from necrotizing enteritis, accompanied by segmental hemorrhagic and necrotizing jejunitis (35). CPB2 toxin is a strongly necrotic and deadly toxin produced by *C. perfringens type C*, which plays an important role in the occurrence and development of piglet diarrhea (36). However, the relationship between piglet diarrhea caused by *C. perfringens type C* infection and m6A methylation modification is rarely studied. In this study, IPEC-J2 cells were treated with CPB2 toxin to construct an *in vitro* model of *C. perfringens type C* infectious piglet diarrhea. MeRIP-seq and RNA-seq technologies were used to determine the presence of m6A-modified mRNAs and lncRNAs, and explore the functions of these m6A-modified mRNAs and lncRNAs, which would provide a reference to analyze piglet diarrhea from the perspective of m6A methylation.

The overall level of m6A methylation is associated with disease. Reducing m6A levels can promote the proliferation of cancer cells, and increasing m6A levels can significantly inhibit tumor development, both *in vivo* and *in vitro* (37). The total modification level of m6A in human lens epithelial cells (HLECs) was increased by high glucose, which promoted HLEC proliferation and inhibited their apoptosis (38). The protective effect of curcumin on lipopolysaccharide (LPS)-induced liver injury and the destruction of liver lipid metabolism might be the result of an increase in m6A RNA methylation (39). Treatment of IPEC-J2 cells with CPB2 toxin can induce apoptosis, inflammation, and barrier function damage (17, 18). In this study, the overall m6A RNA methylation level was significantly upregulated after CPB2 toxin treatment of IPEC-J2 cells, suggesting that m6A methylation is involved in the CPB2 toxin-induced damage to cells.

Many studies related to m6A have been carried out in mammals. For example, 8,306 m6A peaks related to 2,697 genes were detected in the sheep heat stress group, and 12,958 m6A peaks related to 5,494 genes were detected in the control group (40). In the present study, 6,413 different peaks were detected, of which 521 were significantly upregulated and 1,231 were significantly downregulated. From the different peaks, 4,356 mRNAs and 153 lncRNAs with significant differences were identified. M6A is most often found in proximity to stop codons or the 3’ UTR, and has been broadly linked to diverse aspects of mRNA metabolism and function, including altered splicing, decreased mRNA stability, and altered translational efficiency (41). A previous study noted that m6A peaks were enriched in the 3’ UTR and near the stop codons of mature polyadenylated mRNAs (42), which was similar to the results of our study. The m6A sites in the 3’UTR region of human immunodeficiency virus 1 (HIV-1) strongly enhanced the expression of mRNAs by recruiting the cell reader protein YTHDF (43). In this study, m6A peaks, which were mainly enriched in 3’ UTRs, might be involved in regulating the metabolism and function of mRNA.

RRACH is a highly conserved m6A motif in plants and animals (44, 45). RNAs containing the GGACU motif in Hela cell were good substrates for methylases (46). In oligodendrocyte progenitor cells (OPCs), the m6A reader PRRC2A (Proline rich coiled-coil 2 A) binds to the consensus GGACU motif in the Olig2 CDS (coding sequence) in an m6A-dependent manner to stabilize Olig2 mRNA, thus regulating cell proliferation (47). In our sequencing results, the motif sequence GGACU was also significantly enriched. However, whether stability of the RNAs with the GGACU motif site can change the stability of the mRNAs by binding to methylation-related enzymes, and whether they regulate the fate of IPEC-J2 cells induced by CPB2, remains to be further studied.

To explore the role of m6A-modified mRNAs in CPB2 toxin-induced IPEC-J2 cells, we performed statistical analysis of m6A-modified differentially expressed mRNA. We found that whether it was functional enrichment analysis of m6A-modified mRNAs or mRNAs obtained after combined analysis, the Hippo signaling pathway, Wnt signaling pathway and cancer-related signaling pathways were always significantly enriched. These signaling pathways are related to the occurrence of diseases, especially cancer (48, 49). These results indicated that methylated mRNAs play a regulatory role in the pathogenesis of the cells after CPB2 treatment.

To date, there has been no report on the pathway analysis of m6A-modified lncRNAs target genes in IPEC-J2 cells treated with CPB2 toxin. Therefore, in this study, we used GO and KEGG pathway annotation to analyze 153 m6A-modified lncRNA target genes that were significantly differentially expressed in IPEC-J2 cells exposed to 20 μg/mL CPB2 toxin. We attempted to identify the key functions and pathways by which CPB2 treatment may cause IPEC-J2 damage. Comparing the functional enrichment analysis of the significantly different m6A-modified lncRNAs target genes with that of the six lncRNAs from combined analysis showed that the defense response to virus, three pathways of NF-kappa B signaling pathway, cytokine-cytokine receptor interaction, and Influenza A were always significantly enriched. Further analysis revealed that the target gene *CXCL8* coexisted in these three pathways. Target genes such as *MX2* (encoding MX dynamin like GTPase 2), *OAS2* (encoding 2’-5’-oligoadenylate synthetase 2), and *IFIT2* (encoding interferon induced protein with tetratricopeptide repeats 2) were related to the defense response to viruses. MX2 is a cytokine induced by interferon IFN-α, which resists viral infection by inhibiting the accumulation of viral nuclear DNA (50, 51). As a new potentially sensitive biomarker, *OAS2* is significantly up-regulated in psoriasis, a chronic inflammatory systemic disease, and can predict the severity and activity of the disease (52). The antiviral protein IFIT2 was found to be an important limiting factor for rabies virus, which can directly or indirectly resist virus replication (53). In addition, we also found that the three genes *MX2*, *OAS2* and *IFIT2*, which were highly related to the defense response of the virus, were all target genes of lncRNA *ENSSSCG00000042575*. In summary, the differentially methylated lncRNA *ENSSSCG00000042575* might resist virus invasion of cells by regulating target genes related to virus defense. Furthermore, we found that two target genes, *ZC3H12A* (encoding zinc finger CCCH-type containing 12A) and *CXCL8*, are related to immune response activation signal transduction and interleukin-8 receptor binding. ZC3H12A is an essential RNase that prevents immune disorders by directly controlling the stability of a group of inflammatory genes (54). There is compelling evidence suggesting that the m6A modification is particularly important in various pathological and physiological immune responses, including T cell homeostasis and differentiation, inflammation, and type I interferon production (55–58). We speculated that after CPB2 toxin treatment of cells, m6A modification might play an important role in the process of resistance to pathogen invasion by regulating virus defense-related genes, inflammation-related genes, and immune response-related genes.

CXCL8 (also known as interleukin IL-8), an activator of neutrophils, is associated with the initiation and maintenance of inflammation (59). Knockdown of lncRNA MALAT1 inhibited the expression of IL8 and reduced the activation of NF-κB, thereby reducing airway and lung inflammation caused by *Mycoplasma pneumonia* infection (60). In this work, the *CXCL8* gene was enriched for multiple GO terms, such as inflammation, immune response, and cell cycle arrest. Moreover, CXCL8 was also enriched in many pathways, such as the NF-κB signaling pathway, the Toll-like receptor signaling pathway, salmonella infection, and influenza A infection. *IL-8* expression was significantly upregulated in CPB2-treated IPEC-J2 cells (18), and our sequencing results also showed that *CXCL8* was significantly upregulated after CPB2 toxin treatment. Interestingly, we found that *CXCL8* has a targeting relationship with the two lncRNAs *ENSSSCG00000048785* and *ENSSSCG00000048701*, which belong to the group of six significantly differentially expressed m6A-modified lncRNAs identified in this study. The results indicated that m6A-modified lncRNAs might affect the inflammation and immune response of CPB2 toxin-treated cells by regulating the expression of *CXCL8/IL-8*.

In summary, we comprehensively analyzed the m6A methylation profile of RNAs in the control group and the CPB2 group. These results revealed the general characteristics and topological patterns of m6A modification in IPEC-J2 cells, as well as the difference in methylation profiles between the CPB2 group and the control group. Further functional enrichment analysis found that m6A-modified RNAs were related to defense against viruses and inflammation after CPB2 toxin treatment of the cells. The results suggested that three m6A-modified lncRNAs, *ENSSSCG00000048785, ENSSSCG00000042575* and *ENSSSCG00000048701*, are most likely to play a key role in CPB2 toxin-treated IPEC-J2 cells. In conclusion, this study provides a reference for exploring *C. perfringens type C* diarrhea from the perspective of methylation modification.

## Data Availability Statement

The datasets presented in this study can be found in online repositories. The names of the repository/repositories and accession number(s) can be found below: https://www.ncbi.nlm.nih.gov/, SRR15275162-SRR15275173.

## Author Contributions

Conceptualization: JY and SG. Data curation: JZ and XG. Formal analysis: QY. Funding acquisition: QY. Investigation: XH. Methodology: ZY and RL. Project administration: SG. Validation: KX, WW, and JL. Visualization: PW. Writing - original draft: JY. Writing - review and editing: SG. All authors contributed to the article and approved the submitted version.

## Funding

This work was supported by the National Natural Science Foundation of China (31960646), Education Department of Gansu Province: Excellent Graduate student “Innovation Star” project (2021CXZX-351), the Education Science and Technology Innovation Project of Gansu Province, China (GSSYLXM-02), and the Chief Special Project of Pig and Chicken Industry of Gansu Province Modern Agricultural Industrial Technology System (GARS-ZJ-1).

## Conflict of Interest

The authors declare that the research was conducted in the absence of any commercial or financial relationships that could be construed as a potential conflict of interest.

## Publisher’s Note

All claims expressed in this article are solely those of the authors and do not necessarily represent those of their affiliated organizations, or those of the publisher, the editors and the reviewers. Any product that may be evaluated in this article, or claim that may be made by its manufacturer, is not guaranteed or endorsed by the publisher.
