# Supplementary material for: N6-Methyladenosine Methylation Analysis of Long Noncoding RNAs and mRNAs in IPEC-J2 Cells Treated With Clostridium perfringens beta2 Toxin
Source: Front Immunol. 2021 Nov 22;12:769204. doi: 10.3389/fimmu.2021.769204 (PMC8646102; doi:10.3389/fimmu.2021.769204)
Supplement: Supplementary file 1 [file DataSheet_1.zip › Table_1.docx]

Supplementary Table 1 MeRIP-qPCR and RT-qPCR primers for amplifying specific lncRNAs

| GeneId |  | Nucleotide Sequence (5'-3') | Product Length (bp) | Type |
| --- | --- | --- | --- | --- |
| ENSSSCG00000048701 | Forward | AGACTCTGACGTGGTAGGACA | 145 | MeRIP-qPCR /RT-qPCR |
|  | Reverse | TTGGAGAAGTGTCACACCGT |  |  |
| ENSSSCG00000048785 | Forward | CTTTGGACTCTCAAACGCGG | 141 | MeRIP-qPCR / RT-qPCR |
|  | Reverse | AGTGGGTGACAGTGTCTGGA |  |  |
| ENSSSCG00000042575 | Forward | TGAATCAGCAGATACGGGCA | 82 | MeRIP-qPCR / RT-qPCR |
|  | Reverse | GAAACTTGTACGGGCATCCA |  |  |
| ENSSSCG00000042386 | Forward | ACCCAAAGCCTCATCCATCC | 127 | MeRIP-qPCR / RT-qPCR |
|  | Reverse | GAACAACTGCTCAGCATGGC |  |  |
| ENSSSCG00000040169 | Forward | GACGGCGATGGACAGATTGA | 204 | MeRIP-qPCR |
|  | Reverse | TCTGCCCTTTGGTGTCTGTC |  |  |
| ENSSSCG00000045416 | Forward | CGAAGGCATCCCTTTGAGC | 88 | RT-qPCR |
|  | Reverse | GACGAGGGACAGTCTTCATCC |  |  |
| *ENSSSCG00000041817* | Forward | GCGTGGATAGGACCACTCTG | 186 | RT-qPCR |
|  | Reverse | AGGCAACTGGAAGCTACTGC |  |  |
