# Supplementary material for: N6-Methyladenosine Methylation Analysis of Long Noncoding RNAs and mRNAs in IPEC-J2 Cells Treated With Clostridium perfringens beta2 Toxin
Source: Front Immunol. 2021 Nov 22;12:769204. doi: 10.3389/fimmu.2021.769204 (PMC8646102; doi:10.3389/fimmu.2021.769204)
Supplement: Supplementary file 1 [file DataSheet_1.zip › Table_2.docx]

Supplementary Table 2 Summary of reads quality control

| Sample | Raw_Reads | Valid_Reads | Valid% | Q20% | Q30% | GC% |
| --- | --- | --- | --- | --- | --- | --- |
| Control1_IP | 85492316 | 82300026 | 87.19 | 96.52 | 90.94 | 52.87 |
| Control2_IP | 87774992 | 84673100 | 88.09 | 96.52 | 90.97 | 53.28 |
| Control3_IP | 86612186 | 83480922 | 87.76 | 96.33 | 90.59 | 53.39 |
| CPB2_1_IP | 90242216 | 86944976 | 87.80 | 96.56 | 91.01 | 52.96 |
| CPB2_2_IP | 86934932 | 83429842 | 86.82 | 96.39 | 90.78 | 53.41 |
| CPB2_3_IP | 89642446 | 86070416 | 86.79 | 96.41 | 90.77 | 53.99 |
| Control1_input | 97586308 | 93048322 | 84.90 | 96.54 | 91.09 | 55.12 |
| Control2_input | 96994146 | 92000234 | 84.62 | 96.53 | 91.09 | 55.30 |
| Control3_input | 93495320 | 88512114 | 84.20 | 96.30 | 90.67 | 55.92 |
| CPB2_1_input | 95043768 | 90159140 | 84.15 | 96.58 | 91.17 | 55.18 |
| CPB2_2_input | 97478686 | 91769148 | 83.24 | 96.37 | 90.85 | 55.73 |
| CPB2_3_input | 92752804 | 88474062 | 84.76 | 96.46 | 90.95 | 55.71 |
