# Supplementary material for: N6-Methyladenosine Methylation Analysis of Long Noncoding RNAs and mRNAs in IPEC-J2 Cells Treated With Clostridium perfringens beta2 Toxin
Source: Front Immunol. 2021 Nov 22;12:769204. doi: 10.3389/fimmu.2021.769204 (PMC8646102; doi:10.3389/fimmu.2021.769204)
Supplement: Supplementary file 1 [file DataSheet_1.zip › Table_3.docx]

Supplementary Table 3 Summary of reads mapping to the pig reference genome

| Sample | Valid reads | Mapped reads | Unique Mapped reads | Multi Mapped reads |
| --- | --- | --- | --- | --- |
| Control1_IP | 80373652 | 70151859(87.28%) | 55341984(68.86%) | 14809875(18.43%) |
| Control2_IP | 82807672 | 71974281(86.92%) | 56527235(68.26%) | 15447046(18.65%) |
| Control3_IP | 81764254 | 71197784(87.08%) | 55601399(68.00%) | 15596385(19.07%) |
| CPB2_1_IP | 84850966 | 72294412(85.20%) | 58046796(68.41%) | 14247616(16.79%) |
| CPB2_2_IP | 81320858 | 68909160(84.74%) | 55455188(68.19%) | 13453972(16.54%) |
| CPB2_3_IP | 83562226 | 68150139(81.56%) | 55028295(65.85%) | 13121844(15.70%) |
| Control1_input | 86799114 | 63833932(73.54%) | 52095720(60.02%) | 11738212(13.52%) |
| Control2_input | 85711962 | 62707198(73.16%) | 51101051(59.62%) | 11606147(13.54%) |
| Control3_input | 82442168 | 59200245(71.81%) | 47990721(58.21%) | 11209524(13.60%) |
| CPB2_1_input | 83048200 | 58398898(70.32%) | 48164779(58.00%) | 10234119(12.32%) |
| CPB2_2_input | 84294660 | 58281176(69.14%) | 47971041(56.91%) | 10310135(12.23%) |
| CPB2_3_input | 81383998 | 55846823(68.62%) | 45799539(56.28%) | 10047284(12.35%) |
