# Supplementary figures and images for: N6-Methyladenosine Methylation Analysis of Long Noncoding RNAs and mRNAs in IPEC-J2 Cells Treated With Clostridium perfringens beta2 Toxin
Source: Front Immunol. 2021 Nov 22;12:769204. doi: 10.3389/fimmu.2021.769204 (PMC8646102; doi:10.3389/fimmu.2021.769204)

Supplementary Table 4 Refer to the genome to compare the regional distribution.
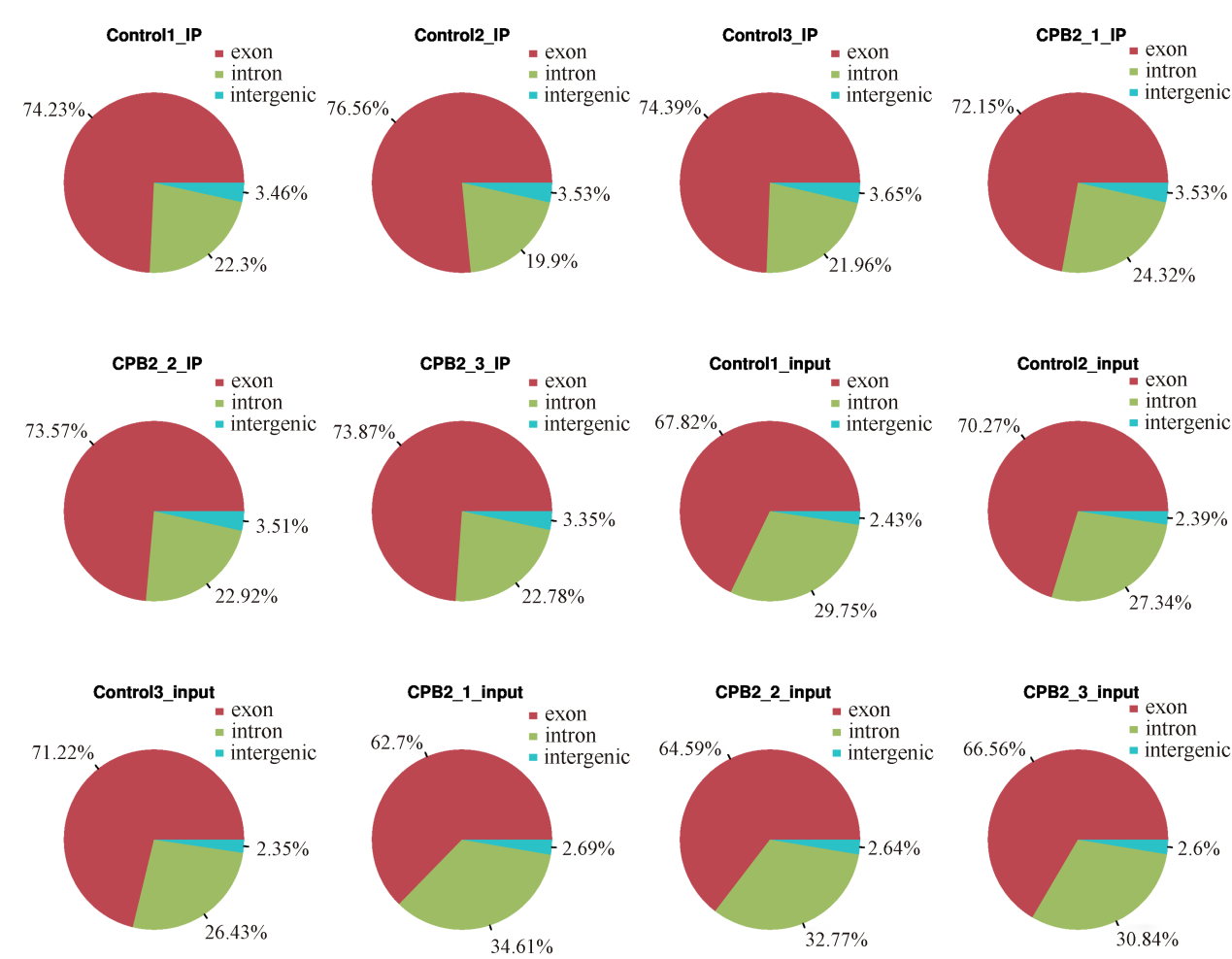

Supplement: Supplementary file 1 [file DataSheet_1.zip › Table_4.docx]
